# Prevention of type 2 diabetes in adults with impaired glucose tolerance: the European Diabetes Prevention RCT in Newcastle upon Tyne, UK

**DOI:** 10.1186/1471-2458-9-342

**Published:** 2009-09-16

**Authors:** Linda Penn, Martin White, John Oldroyd, Mark Walker, K George MM Alberti, John C Mathers

**Affiliations:** 1 Public Health Research Programme, Institute of Health and Society, Newcastle University, Newcastle upon Tyne, NE24HH, UK; 2 Human Nutrition Research Centre, Institute for Ageing and Health, Newcastle University, Newcastle upon Tyne, NE24HH, UK; 3 Diabetes Research Group, Newcastle University, Newcastle upon Tyne, NE24HH, UK; 4 Medicine, Nursing and Health Sciences, Monash University, Victoria 3800, Australia; 5 Institute of Cellular Medicine, Newcastle University, Newcastle upon Tyne, NE24HH, UK; 6 Department of Endocrinology and Metabolism, St Mary's Hospital and Imperial College, University of London, London, W2 1NY, UK

## Abstract

**Background:**

Diabetes prevalence is increasing. The Finnish Diabetes Prevention Study (DPS) showed a 58% reduction in Type 2 Diabetes (T2D) incidence in adults with impaired glucose tolerance (IGT). The European Diabetes Prevention Study (EDIPS) extends the DPS to different European populations, using the same study design. In the Newcastle arm of this study (EDIPS-Newcastle), we tested the hypothesis that T2D can be prevented by lifestyle intervention and explored secondary outcomes in relation to diabetes incidence.

**Methods:**

We recruited 102 participants (42 men and 60 women, mean age 57 years, mean BMI 34 kgm^-2^) with IGT to EDIPS-Newcastle and randomised to Intervention and usual care Control groups. The intervention included individual motivational interviewing aimed at: weight reduction, increase in physical activity, fibre and carbohydrate intake and reduction of fat intake (secondary outcomes). The primary outcome was diagnosis of T2D.

**Results:**

Mean duration of follow-up was 3.1 years. T2D was diagnosed in 16 participants (I = 5, C = 11). Absolute incidence of T2D was 32.7 per 1000 person-years in the Intervention-group and 67.1 per 1000 person-years in the Control-group. The overall incidence of diabetes was reduced by 55% in the Intervention-group, compared with the Control-group: RR 0.45 (95%CI 0.2 to 1.2).

Explanatory survival analysis of secondary outcomes showed that those who sustained beneficial changes for two or more years reduced their risk of developing T2D.

**Conclusion:**

Our results are consistent with other diabetes prevention trials. This study was designed as part of a larger study and although the sample size limits statistical significance, the results contribute to the evidence that T2D can be prevented by lifestyle changes in adults with IGT. In explanatory analysis small sustained beneficial changes in weight, physical activity or dietary factors were associated with reduction in T2D incidence.

**Trial Registration:**

International Standard Randomised Controlled Trial Number registry (ISRCTN)

Registry number: ISRCTN 15670600

## Background

The prevalence of type 2 diabetes (T2D) is increasing rapidly and there are causal associations with obesity, diet and physical inactivity[1]. In the UK almost 5% of people have T2D and treatment costs absorb a high proportion of the health care budget[2]. Type 2 diabetes affects both quality of life and mortality and is a growing public health challenge.

Type 2 diabetes is a progressive metabolic disease with impaired glucose tolerance (IGT) as an early stage in disease development [3]. Several large, well-designed trials with long-term follow-up, evaluating interventions to prevent the onset of diabetes in people with IGT have been published [4-8]. The Finnish Diabetes Prevention Study (DPS) showed a 58% reduction in T2D incidence following lifestyle intervention in adults with IGT[8]. The European Diabetes Prevention Study (EDIPS) extends the DPS to different European populations, using a similar study design[9,10]. The other EDIPS centres, in addition to Finland and Newcastle, are in Maastricht, the Netherlands and Sardinia, Italy.

The EDIPS in Newcastle upon Tyne, UK (EDIPS-Newcastle) was designed to contribute to the evidence for diabetes prevention by lifestyle modification in people with IGT. In this paper, we describe the methods and report both pragmatic and explanatory analyses of EDIPS-Newcastle in relation to diabetes prevention.

## Methods

### Ethics statement

The Newcastle and North Tyneside NHS Research Ethics Committee approved the study protocol and all participants gave informed, written consent before the start of the study.

### Study design, randomisation and end points

We conducted a Randomised Controlled Trial (RCT) with one Intervention and one Control arm. Participants were randomly allocated either to intensive behavioural interventions to promote dietary modification and increased physical activity or to a minimal intervention Control group. The planned maximum follow-up for any individual was five years.

Recruitment was by referral from primary care physicians who identified eligible people likely to be at risk of impaired glucose regulation (using the criteria: aged over 40 and overweight (BMI > 25 kgm^-2^)) from their primary care databases and invited them to participate.

Oral glucose tolerance tests (OGTT) were conducted in the Clinical Research Facility, Royal Victoria Infirmary Newcastle upon Tyne. Eligible participants (with IGT) were randomly allocated to the Intervention (I) or Control (C) group using randomisation lists, prepared independently by the EDIPS co-ordinating centre in Helsinki. Randomisation was stratified by sex and by two hour plasma glucose value (derived from the mean of two standard oral glucose tolerance tests (OGTTs) - stratum 1: 7.8 to 9.4 mmol/l; stratum 2: 9.5 to 11.1 mmol/l). Blinding of participants and intervention staff was not possible. Data collection staff were blinded to the extent that this was possible given participants' knowledge of their allocation.

### Outcomes

1. Development of T2D, diagnosed on the basis of two OGTTs conducted with 1-12 weeks of each other, assessed annually from baseline, was the primary study end point.

2. Other end points were myocardial infarction or sudden cardiac death, intermittent claudication, stroke or death from other causes.

3. Secondary outcomes were changes in BMI (kgm^-2^), intakes of carbohydrate and fat (as percentages of total energy intake) and dietary fibre (g), and participation in physical activity (minutes of moderate aerobic physical activity per day).

### Inclusion and exclusion criteria

We included people aged over 40 years with BMI > 25 kgm^-2 ^and with established IGT defined as a mean 2-hour plasma glucose value ≥ 7.8 mmol/l and < 11.1 mmol/l from two consecutive standard OGTTs (glucose load 75 g) conducted between one and 12 weeks apart (World Health Organisation 1999 classification)[11]. If the 2-h OGTT value was just over the diabetes threshold (11.1-11.5 mmol/l) or under the IGT threshold (7.3-7.7 mmol/l), a second OGTT was performed within 1-12 weeks. If the mean of the 2-h values from the two OGTTs was ≥ 7.8 and <11.1 mmol/l the individual was eligible for inclusion. A diabetic value in the second OGTT was an exclusion criterion, even if the mean value was in the IGT range. People with previous diagnosis of diabetes, or with chronic illness that would make participation in moderate physical activity impossible, or on a special diet for medical reasons were excluded.

### Measurements

All participants received a clinical assessment prior to randomisation and annually thereafter, including an OGTT, anthropometric and blood biochemistry measurements. Additionally they were asked to complete a health status questionnaire (RAND-36),[12] the WHO cardiovascular questionnaire[13] and annual three-day (two week days and one weekend day) diet and physical activity diaries.

Assessments were conducted in the Clinical Research Facility, Royal Victoria Infirmary in Newcastle upon Tyne.

Body weight was measured to the nearest 0.1 kg in light indoor clothing using SECA 770 electronic scales (Alpha Model 770, SECA Limited, Birmingham, UK). Height was measured to the nearest half centimetre using a SECA 225 stadiometer (SECA Limited, Birmingham, UK). Waist circumference was measured to the nearest centimetre at the midpoint between the iliac crest and the lower rib margin. Percentage body fat was measured by bioelectrical impedance, using a BODYSTAT 1500 (BODYSTAT Ltd, Douglas, Isle of Man, UK).

Blood was collected from the antecubital vein with the participant in a sitting position using a needle to insert a cannula. If a tourniquet was used it was opened immediately after the needle had entered the vein. Glucose was measured in venous plasma, using a Yellow Springs glucose analyser (Yellow Springs Instrument co Inc, Ohio, USA.). Food portion sizes were validated by the study dietician using a photographic food atlas [14] and nutrient composition was analysed using Microdiet software (Downlee Systems, Salford, UK). The activity diary covered the whole 24 hour period on all three days. Participants were asked to record activity for each 30 minute period throughout the day starting from midnight (midnight to 00.30, 00.30 to 1.00, 1.00 to 1.30 etc.) using an integer scoring system based on MET scores. For example, lying down was scored 1 and brisk walking was scored 6. A 24-hour activity score of 80 would be achieved by lying down for eight hours and sitting for the rest of the day. Thirty minutes of brisk walking would add four to a participant's score on any day.

### Interventions

Behavioural interventions consisted of regular individual advice from a dietician and physiotherapist trained in motivational interviewing [15]. Intervention participants were also invited to some group sessions, notably 'cook and eat' events. They also received a regular quarterly newsletter. The newsletter contained: healthy eating recipes, nutritional information, suggestions for local walks, and exercise options. The dietary intervention provided advice and counselling to develop an individual plan for behaviour change, with the aim of achieving: >50% total dietary energy intake from carbohydrate, reduced total and saturated fat intake with <30% total dietary energy from fat, increased fibre intake, and weight loss to achieve BMI <25 kgm^-2^[16]. Analysis of participants' three day food diaries, collected quarterly, and regular weight and waist measurements were used to tailor individual dietary advice. The physical activity intervention was designed to encourage participation in increased physical activity equivalent to accumulating 30 minutes of moderate aerobic physical activity per day. Analysis of participants' three day activity diaries, collected quarterly, was used in motivational feedback and to tailor goals for increasing physical activity, which were negotiated at each visit.

Participants in the Intervention group were seen by the intervention team (dietician and physiotherapist) for approximately 30 minutes per session, immediately following randomisation and two weeks later, then monthly for the first three months and every three months thereafter up to five years. In addition to individual and group activities, participants received an information pack detailing facilities and opportunities for physical activity in Newcastle upon Tyne, a City Card (a discount scheme run by Newcastle Leisure Services offering up to 80% discount on access to physical activity facilities) and the opportunity to meet with a trainer at a local leisure centre and take part in an induction session. Information generated from earlier studies in Newcastle was used to tailor the intervention to the local conditions [17-19]

### Control condition

Both Intervention and Control groups were offered standard health promotion advice including widely available contemporary written leaflets on healthy eating and physical activity. Control group participants were otherwise offered 'usual care' by their primary care physician.

### Sample size

EDIPS-Newcastle was designed to contribute to the European study. We aimed for a sample size of 100 participants (50 in each arm), contributing to a planned total of 750 participants across Europe.

### Analysis

The Statistical Package for Social Scientists (SPSS inc. version 15) was used for analyses. We used independent t-tests to compare continuous variables and Chi-squared tests to compare categorical variables in the Intervention and Control groups at baseline. Pragmatic (intention-to-treat) analysis of the primary endpoint was conducted using Kaplan-Meier survival analysis to determine the difference in relative risk of cumulative incidence of diabetes between the Intervention and Control groups.

For secondary outcomes we used independent t-tests to compare the Intervention and Control group means of continuous variables at baseline and in each year of the study.

For the explanatory analyses of secondary outcomes, we pooled the Intervention and Control groups and considered each secondary outcome measure in turn. For these outcomes we used a scoring system whereby any individual's beneficial change from their baseline value in an outcome measure was scored 1 for each year of beneficial change (cut off change values were 0.01 for beneficial increase or -0.01 for beneficial reduction) and all other values (no change or detrimental change) were scored 0. The scores for each participant were totalled across study years and the participants were divided into two groups (for each measure separately): a 'sustained change' group with a score of two or more, indicating beneficial change in the parameter for at least two years; and a 'no sustained change' group with a score less than 2, indicating less than two years beneficial change, no change or detrimental change. These groups were then compared using Kaplan-Meier survival analysis for progression to T2D. We also used independent t-tests to compare the 'sustained change' and 'no sustained change' group means and the difference between the groups for each of the secondary outcomes at baseline and in each year of the study. Two years of sustained change was chosen as the criterion for explanatory analysis groups after consideration of other possibilities (e.g. one year or three years) and with reference to the findings of our qualitative study linked to this trial[20].

The baseline characteristics, weight, height, BMI, waist circumference, hip circumference, body fat %, plasma glucose (fasting, 30 minute, 60 minute and 120 minute), plasma insulin (fasting, 30 minute and 120 minute), age, sex, socioeconomic status and working capacity of the 'sustained change' and 'no sustained change' groups for each secondary outcome were compared for equality with t-tests or Chi-squared tests as appropriate.

## Results

### Recruitment

**Figure 1:**
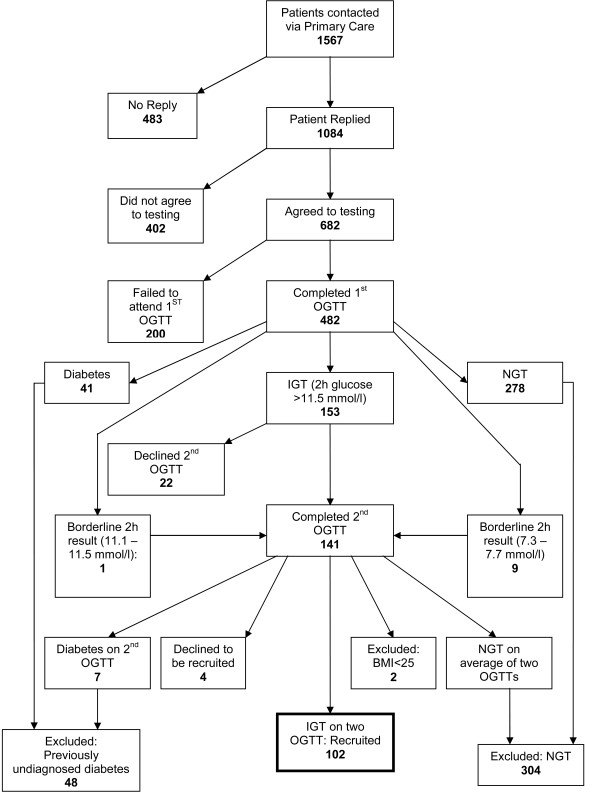
**Flow of participants during recruitment to EDIPS-Newcastle**.

**Figure 2:**
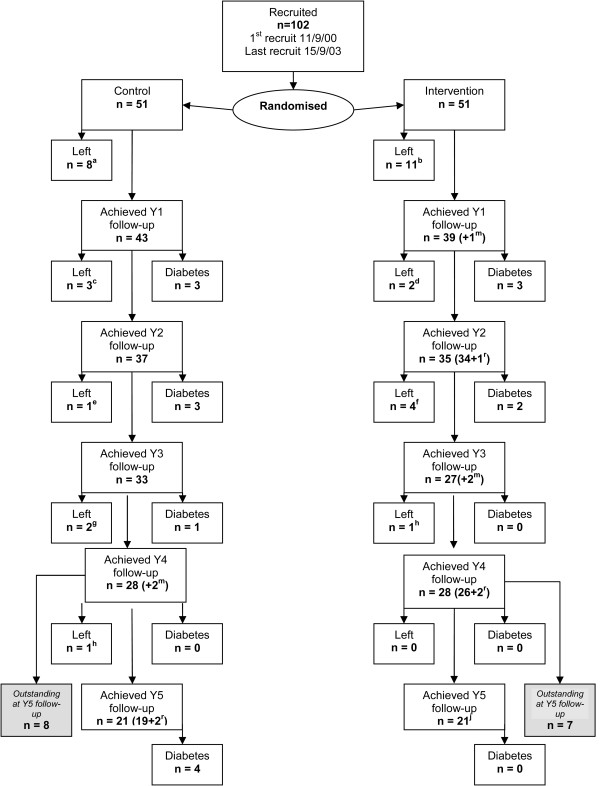
**Flow of participants through EDIPS-Newcastle RCT**. ^m^ participants who missed an annual review, but returned the following year (counted in for the survival analysis). ^r ^participants returning after missing an annual review  Reasons for leaving the trial:  ^a^ lack of time=4, physician diagnosis=1, illness=3 (1 of these died of colon cancer a year later)  ^b^ lack of time=5, changed mind=2, physician diagnosis=1, not known=2, died=1 ^c^ not known=2, mother died=1  ^d^ not known=1, family bereavement=1 ^e^not known=1 ^f^lack of time=1, not known=1, illness=1, back surgery=1 ^g ^lack of time=1, illness=1^h ^lack of time=1^i ^lack of time=1^j ^1= completed to year 5 and died later of lung cancer

We recruited 102 participants to the study and they were randomised in equal numbers to the Intervention and Control groups. Recruitment and trial progression are shown in Figures 1 and 2.

### Progression and trial profile

In the first year 19 participants (I = 11, C = 8) left the study. The mean age of those who left in the first year was 54.8(95% CI: 49.3 to 60.4) years compared with 57.2 years (95% CI: 55.5 to 59.8) for those who stayed in the study.

### Comparison of Intervention and Control groups at baseline

There was little difference in any of the anthropometric, clinical, social or demographic characteristics of the two groups measured at baseline (Tables 1 and 2). Participants were taking a range of drugs, including statins, beta blockers, anti-inflammatory medication and ACE inhibitors. There were no significant differences in medication between Intervention and Control groups.

**Table 1:** Baseline characteristics: mean (SD) for continuous variables by trial group.

**Measurement**	**Intervention (n = 51)**	**Control (n = 51)**
BMI (kgm^-2^)	34.1 (5.5)	33.5 (4.6)
Waist (cm)	104.6 (11.3)	104.3 (9.2)
Hip (cm)	111.0 (11.7)	110.3 (9.0)
Weight (kg)	93.4 (16.0)	90.6 (12.5)
Height (cm)	165.5 (8.9)	164.9 (10.2)
Body fat %	40.2*(9.4)	40.1 (9.9)
Plasma glucose (mmol/l)		
Fasting	5.7 (0.6)	5.8 (0.5)
30 minute	9.9 (1.3)	9.8*(0.9)
60 minute	11.5 (1.9)	11.5*(1.6)
120 minute	8.7 (1.1)	8.9 (1.3)
Plasma insulin (mU/l)		
Fasting	16.9 (12.4)	17.3 (7.4)
30 minute	97.5 (47.1)	85.08 (40.4)
120 minute	118.0 (58.0)	122.18 (55.3)

*n = 49

**Table 2:** Baseline characteristics: number (%) by trial group for demographic variables.

	**Intervention (n = 51)**	**Control (n = 51)**
**Age [mean (range)]**	56.8 (40-72)	57.4 (38-74)
**Sex**		
Male	21 (41.2)	20 (39.2)
Female	30 (58.8)	31 (60.8)
**Current working capacity**		
Retired	24 (47.1)	23 (54.8)
Full working capacity	18 (35.3)	17 (40.5)
Unable to work	6 (11.8)	0
Data unavailable	3 (5.9)	2 (4.8)
**Socio-economic status by type of work**		
Manual	23 (45.1)	26 (51.0)
Non-manual	19 (37.3)	19 (37.3)
Data unavailable	9 (17.6)	6 (11.8)

### Main outcomes

**Figure 3:**
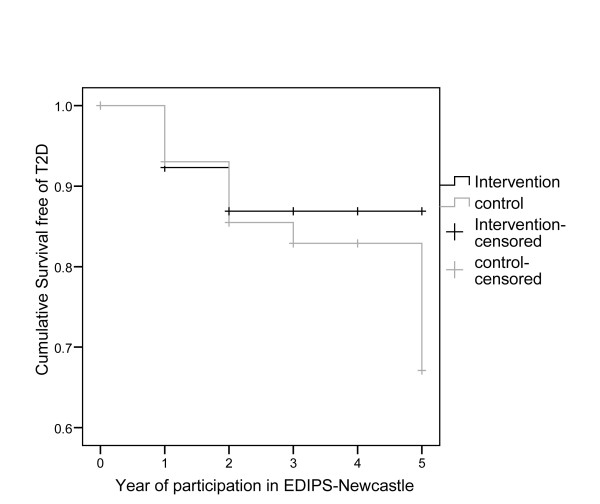
**Progression to type 2 diabetes by trial group**.

Mean duration of follow up was 3.11 years (range 0 to 5). T2D was diagnosed in a total of 16 participants (I = 5, C = 11). The absolute incidence of T2D was 32.7 (95% CI: 10.7 to 74.6) per 1000 person years of follow-up in the Intervention group and 67.1 (95% CI: 34.2 to 117.5) per 1000 person years of follow-up in the Control group. The relative risk of T2D in the Intervention group, compared with the Control group was 0.45 (95% CI: 0.2 to 1.2). After year two of follow-up, there were no further incidences of T2D in the Intervention group. Thus, overall the cumulative incidence of diabetes was 55% less in the Intervention group compared with the Control group. Kaplan-Maier Survival analysis for Intervention and Control groups is shown in Figure 3.

A post study power calculation showed that 51 per group provided 37% power to detect the difference found (80% power would have required149 per group, a number that will be exceeded in pooled analyses with other EDIPS centres).

If participants who left the trial and were later reported to have developed T2D by their physician were included in the analysis as having developed diabetes (rather than having left), then the number of cases of diabetes becomes 20 (I = 7, C = 13) and the relative risk of diabetes incidence becomes 0.54 (95% CI:0.2 to 1.2).

### Secondary outcomes

There were no significant differences in mean values for secondary outcome measures between the Intervention and Control groups at baseline or at annual follow-up in any year. Analysis of difference from baseline value in these secondary outcome measures showed a significant difference between Intervention and Control groups in weight loss at year 1 follow-up only (mean weight change: Intervention group = -2.3 kg, Control group = 0.01 kg; mean difference -2.5 (95% CI: -4.2 to 0.7)kg, p = 0.007). Only three participants achieved BMI < 25 kgm^-2^.

Post-hoc analysis of reversion to normal glucose tolerance showed no difference between the intervention and the control groups either for reversion to normal glucose tolerance on at least one occasion (I = 23, C = 22: RR 1.1 95%CI 0.7 to 1.6), or for reversion to normal glucose tolerance on two consecutive occasions (I = 17, C = 11: RR 1.5 95% CI 0.8 to 3.0).

### Explanatory analysis of secondary outcomes

**Figure 4:**
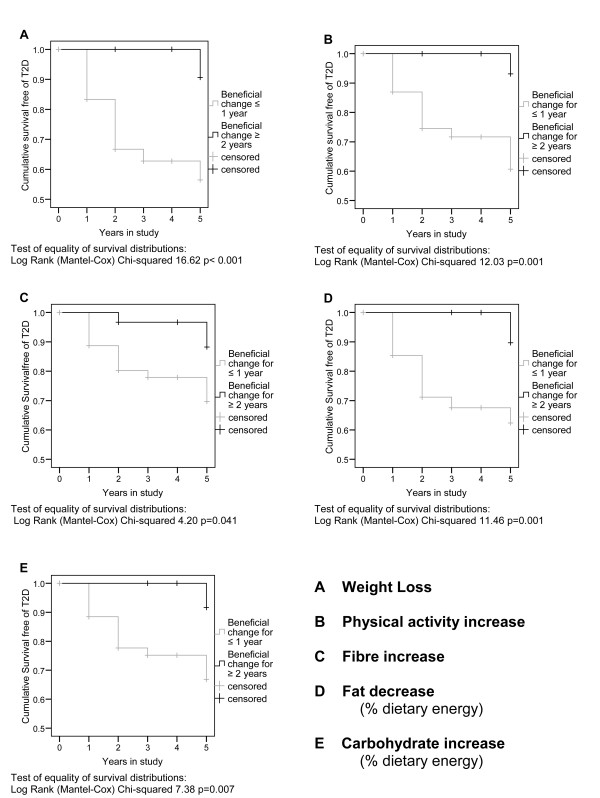
**Relationship between changes in secondary outcomes and progression to T2D in trial participants (trial groups pooled)**. This figure shows the results of survival analysis based on beneficial change in secondary outcome measures maintained for two or more years. Intervention and control group data was pooled for this analysis.

**Figure 5:**
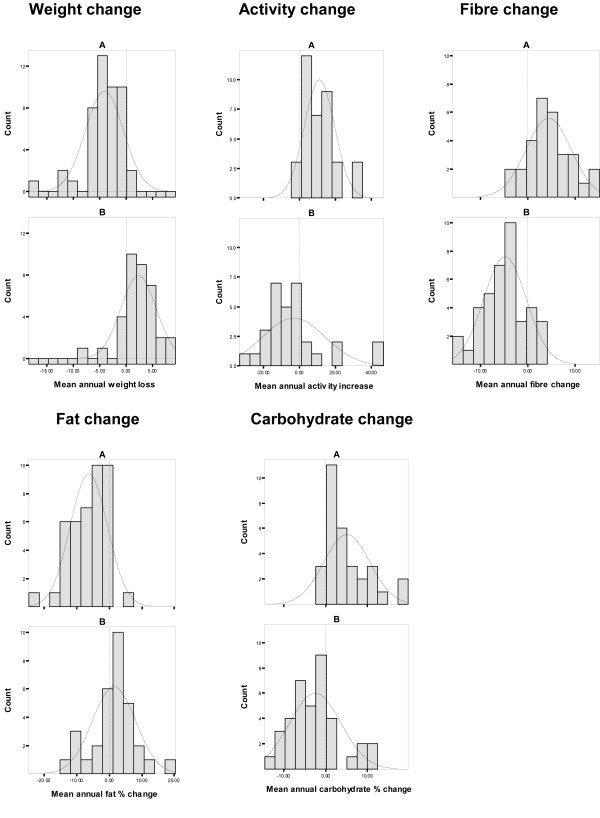
**Mean annual changes in secondary outcome measures by sustained beneficial change (A) and no sustained beneficial change (B) groups**. This figure shows the range and distribution of change in secondary outcome measures in the two groups defined by A: 'beneficial direction of change in an outcome measure sustained for two or more years' and B: no beneficial direction of change in the outcome measure. Intervention and control group data was pooled for this analysis.

The results of the explanatory survival analyses are shown in Tables 3 and 4 and Figures 4 and 5.

**Table 3:** Relationship between sustained change* in secondary outcomes and progression to T2D by trial group.

	**Number in intervention group (%)**	**Number in control group (%)**	**Total number (%)**	**Number of cases of T2D over five years follow-up**
**Body weight**				
Sustained beneficial change	23 (45)	24 (47)	47 (46)	3
No sustained beneficial change	28 (55)	27 (53)	55 (54)	13
				
**Physical activity score**				
Sustained beneficial change	18 (35)	19 (37)	37 (36)	2
No sustained beneficial change	33 (65)	32 (63)	65 (64)	14
				
**Intake of dietary fibre**				
Sustained beneficial change	15 (29)	15 (29)	30 (29)	3
No sustained beneficial change	36 (71)	36 (71)	72 (71)	13
				
**% energy intake from fat**				
Sustained beneficial change	21 (41)	21 (41)	42 (41)	3
No sustained beneficial change	30 (59)	30 (59)	60 (59)	13
				
**% energy intake from carbohydrate**				
Sustained beneficial change	15 (29)	16 (31)	31 (30)	2
No sustained beneficial change	36 (71)	35 (69)	71 (70)	14

* Sustained beneficial change in secondary outcome measures was defined as: a beneficial change (>0.01 units) maintained for two or more years (i.e. weight loss, reduction in % energy intake from fat, increase in % intake from carbohydrate, increase in intake of dietary fibre and increase in physical activity score).

**Table 4:** Mean (SD) values of secondary outcomes: comparison of sustained beneficial change* and no sustained beneficial change groups in each study year.

**Outcome**	**Time**		**Sustained beneficial change**		**No sustained beneficial change**	**Difference**	**p-value**
		**n**	**Mean (SD)**	**n**	**Mean (SD)**	**Mean (95% CI)**	
**Weight (Kg)**							
	Year 0	47	91.4 (12.8)	55	92.6 (15.6)	-1.2 (-6.9, 4.5)	0.68
	Year 1	47	88.4 (14.7)	35	93.0 (13.8)	-4.6 (-10.8, 1.6)	0.15
	Year 2	47	87.0 (13.7)	25	93.5 (17.0)	-6.5 (-13.8, 0.9)	0.08
	Year 3	43	86.7 (13.4)	17	87.4 (9.2)	-0.7 (-7.8, 6.4)	0.84
	Year 4	43	87.9 (13.7)	13	88.0 (10.1)	-0.1 (-8.3, 8.2)	0.99
	Year 5	32	88.4 (14.2)	10	85.1 (11.7)	3.3 (-6.8, 13.3)	0.52
**Activity(score/day)**							
	Year 0	37	93.6 (8.3)	49	103.1 (16.7)	-9.5 (-15.4,-3.5)	0.002
	Year 1	33	101.0 (12.8)	26	100.9 (14.0)	0.1 (-6.9, 7.07)	0.99
	Year 2	31	102.5 (12.4)	18	96.6 (14.1)	6.0 (-1.8, 13.8)	0.13
	Year 3	33	106.4 (17.0)	18	100.6 (11.2)	5.8 (-3.1, 14.7)	0.20
	Year 4	34	105.3 (11.6)	13	97.9 (12.6)	7.5 (-0.3, 15.2)	0.06
	Year 5	22	103.7 (15.5)	10	103.2 (11.5)	0.4 (-10.8, 11.7)	0.94
**Fibre (g/day)**							
	Year 0	30	17.6 (6.1)	54	20.5 (6.5)	-2.9 (-5.8, -0.0)	0.050
	Year 1	29	22.4 (10.2)	37	16.2 (6.3)	6.2 (2.2, 10.3)	0.003
	Year 2	27	24.4 (10.0)	32	17.8 (6.8)	6.6 (2.2, 11.0)	0.004
	Year 3	27	21.2 (8.1)	26	15.7 (5.3)	5.5 (1.7, 9.3)	0.005
	Year 4	24	21.5 (7.9)	22	16.7 (7.4)	4.7 (0.2, 9.3)	0.043
	Year 5	20	20.6 (8.2)	13	15.1 (4.9)	5.4 (0.3, 10.6)	0.039
**Fat (% energy)**							
	Year 0	42	37 (07)	51	31 (09)	6.2 (2.6, 9.7)	0.001
	Year 1	39	31 (10)	27	34 (06)	-2.2 (6.5, 2.1)	0.306
	Year 2	37	31 (06)	22	35 (06)	-4.4 (7.8, -1.0)	0.011
	Year 3	41	29 (07)	12	36 (07)	-6.2 (-1.1, -1.7)	0.007
	Year 4	36	29 (06)	10	35 (06)	-5.2 (-9.5, -0.9)	0.02
	Year 5	24	30 (07)	9	38 (05)	-8.3 (-13.5, -3.1)	0.003
**Carbohydrate (% energy)**							
	Year 0	31	44(07)	52	49(08)	-4.5(-8.1,1.0)	0.013
	Year 1	28	48(11)	38	48(10)	-0.3(-5.4,4.6)	0.89
	Year 2	26	50(07)	33	46(10)	3.3(-1.2, 7.9)	0.15
	Year 3	31	52(08)	22	44(11)	8.4(3.1, 13.7)	0.002
	Year 4	28	51(10)	18	47(08)	3.5(-2.1, 9.1)	0.22
	Year 5	21	48(09)	12	47(07)	1.0(-5.2, 7.2)	0.74

* Sustained beneficial change in secondary outcome measures was defined as: a beneficial change (>0.01 units) maintained for two or more years (i.e. weight loss, reduction in % energy intake from fat, increase in % intake from carbohydrate, increase in intake of dietary fibre and increase in physical activity score).

There was no significant difference at baseline between the 'sustained change' and 'no sustained change' groups defined by any of the secondary outcomes for the characteristics compared.

Explanatory survival analysis of secondary outcome measures showed that, irrespective of randomisation allocation, groups of individuals who sustained beneficial direction of change in secondary outcomes for two or more years reduced significantly their risk of developing T2D compared with others in the study. Kaplan-Meier Log Rank, Chi-Square results: weight 16.6 (p < 0.001), physical activity 12.0 (p = 0.001), percentage carbohydrate intake 7.4 (p = 0.007), percentage fat intake 11.5 (p = 0.001), or total fibre intake 4.2 (p = 0.041) (Figure 4).

There were almost equal contributions from the intervention and control allocations to each 'sustained change' group of individuals (Table 3)

The mean values for secondary outcomes were significantly different at baseline for 'sustained beneficial change' and 'no sustained beneficial change' groups in each of the secondary outcome measures except body weight. In each case the 'sustained change' group had a significantly less beneficial baseline value (Table 4). The sustained change group had significantly more beneficial values for percentage energy intake from fat from year 2 onwards and for fibre intake from year 1 onwards. The distribution of change, based on mean annual values, in the sustained change and no sustained change groups for each secondary outcome measure are shown in Figure 5 and Table 5.

**Table 5:** Mean (SD) of all years' annual change in secondary outcome measures: comparisonbetween sustained beneficial change* and no sustained beneficial change groups.

**Outcome**		**Sustained beneficial change**		**No sustained beneficial change**	**Difference**	**p-value**
	**n**	**mean (SD)**	**n**	**mean (SD)**	**mean (95% CI)**	
Weight (Kg)	47	-4.1 (3.6)	36	2.3 (3.3)	-6.4 (-8.0, -4.9)	<.001
Activity(score/day)	37	11.1 (8.4)	31	-3.2 (17.5)	14.3 (7.8, 20.8)	<.001
Fibre intake (g/day)	30	4.4 (4.7)	39	-4.8 (4.5)	9.2 (7.0, 11.5)	<.001
Fat (% energy)	42	-6.3 (5.7)	32	1.4 (6.7)	-7.7 (-10.6, -4.8)	<.001
Carbohydrate (% energy)	31	5.0 (5.5)	38	-2.5 (6.2)	7.5 (4.7, 10.4)	<.001

* Sustained beneficial change in secondary outcome measures was defined as: a beneficial change (>0.01 units) maintained for two or more years (i.e. weight loss, reduction in % energy intake from fat, increase in % intake from carbohydrate, increase in intake of dietary fibre and increase in physical activity score).

## Discussion

### Summary of main findings

The results of the EDIPS-Newcastle suggest that progression to T2D can be prevented or delayed by interventions designed to change the lifestyles of participants with IGT. The overall incidence of diabetes was reduced by 55% in the Intervention group compared with the Control group, a magnitude of effect similar to that seen in the other main diabetes prevention trials [4-8]. However this study was designed as part of a larger study and was not powered for statistical significance.

Explanatory analysis highlights the efficacy of weight loss and of maintaining beneficial changes in diet and physical activity. The baseline differences in diet and physical activity measures suggest that those with the greatest capacity for beneficial change at baseline (because they were furthest from the healthy targets) were most able to sustain and to benefit from the changes made. The annual changes (means and distributions) in the groups that did and did not sustain beneficial change, demonstrate that small amounts of sustained change are effective in reducing risk.

### Strengths and limitations

Our study built on the highly successful Finnish DPS, replicating its methods closely. Participants were recruited following two consecutive OGTTs taken between one and 12 weeks apart, providing strong evidence of IGT at baseline. The interventions were adapted for the UK cultural context, but we retained the same intervention targets for participants. A major strength was the length of follow-up (mean 3.1 years), equivalent to that achieved in the other main diabetes prevention trials [4-8].

As with any pragmatic trial, there were some limitations. EDIPS-Newcastle was planned as part of a larger study and was not powered to measure significant changes in incidence of T2D or secondary outcomes. Nevertheless, the results accord with previous trials and add to the existing evidence that progression to T2D can be prevented or delayed by lifestyle changes. Data will be pooled with those from other EDIPS collaborating centres for substantive analyses. Recruitment took longer than expected (18 months instead of 6 months), which meant that, despite an extension to the study period, we were unable to follow-up 15 cases at five years. Some of the problems in recruiting and maintaining the study sample are highlighted in Figures 1 and 2. Of the 141 people who received a second OGTT, 26 (18%) were found to have reverted to NGT, more people than anticipated left the study early, especially in the first year for various reasons, and some participants failed to complete the baseline data collection.

In the explanatory analyses, we compared individual change in secondary outcomes with each participant's own baseline data. This had the advantages both of measuring the direction of individual change, which we classified as beneficial or not irrespective of whether the participant had achieved the intervention target (e.g. >50% energy intake from carbohydrate), and taking account of individual variations in reporting (e.g. of dietary intake).

### Meaning and implications of the findings

Our findings for the primary outcome concur well with previous trials and indicate >50% reduction in risk of development of T2D is achievable in those with IGT when randomised to a lifestyle intervention [4-8]. In addition the results presented here, together with an associated qualitative study, [20] led us to investigate the effects of sustained beneficial behavioural change among some members of the Control group (in addition to participants in the Intervention group) and to determine the importance of maintaining change in the prevention of T2D. The explanatory analysis demonstrated the efficacy of sustained lifestyle changes, where these were measured as individual beneficial change.

Whilst the control treatment was 'minimal intervention', it involved an annual clinical review and annual food intake and physical activity diaries. It has been recognised that a diagnosis of IGT, together with annual clinical reviews, constitute rather more than 'usual care'[21]. The motivational effect of monitoring needs to be assessed separately and considered in future pragmatic trials.

The problem of identifying persistent IGT for trial recruitment has been partly addressed since we commenced this trial by the development of prospective diabetes risk scores, such as FINDRISC [22]. Risk scores could also reduce the potential for selection bias when participants are recruited through primary care. Large trials, where the participants have been recruited on the basis of risk scores, are underway [23]. However risk scores do not diagnose T2D nor monitor progression, so the OGTT is likely to remain the diagnostic test of choice in future trials.

Different people require different motivational input to achieve behavioural change: for some, risk identification at baseline is decisive; others need continual support to change behaviour. In the Intervention group, engagement with the intervention varied from those participants who remained in the trial but returned only for the clinical reviews and those who attended all opportunities. In addition we were aware that some Control group participants sought help from elsewhere (e.g. weight loss groups). Future research should identify sub-groups with different levels of motivation for targeting with more or less intensive interventions.

Compared with the Finnish DPS, EDIPS-Newcastle had a larger proportion of participants who left the trial in the first year. In Finland much primary health care, including health promotion, is delivered via occupational health. This is not the case in the UK [24]. Some of our participants took a day from their employment annual leave allowance to attend their clinical review. Finding ways of improving the accessibility of intervention opportunities is an important area for further research.

Future pragmatic trials of the efficacy of lifestyle intervention in T2D prevention should address: achieving higher levels of recruitment; acceptable, ethical and efficient data collection tools; and acceptable, safe and efficient monitoring schemes to evaluate trial progression. This may involve investigating more convenient times and locations for intervention delivery, including workplaces. Refining the physical activity and dietary advice within T2D prevention interventions to maximise initiation, magnitude and maintenance of change remains a continuing challenge and should be the subject of further investigation.

Diabetes prevention policies and management programmes are currently being implemented in some European countries, notably in Finland and Germany, and European guidelines for the primary prevention of T2D are being developed[25,26]

EDIPS-Newcastle was planned as part of a larger study. Subsequent analysis of data from the combined European study centres will further illuminate diabetes prevention.

## Conclusion

In conclusion: the results of the Newcastle arm of the European Diabetes Prevention Study are consistent with those of other diabetes prevention trials. This study was designed as part of a larger study and although the sample size limits statistical significance, the results contribute to the evidence that T2D can be prevented by lifestyle changes in adults with IGT. In explanatory analysis we showed that small sustained beneficial changes in secondary outcome measures: weight loss, increase in physical activity, reduction in dietary energy intake, reduction in percentage fat intake and increase in percentage fibre intake, were associated with reduction in T2D incidence.

## Competing interests

The authors declare that they have no competing interests.

## Authors' contributions

MWh, JO, JCM and KGMMA designed the study, based on the Finnish DPS protocol, and MWh, JCM and KGMMA secured funding. MWa provided clinical advice. JO managed the fieldwork in year 1. LP managed the fieldwork from year 2, collated and analysed the data and, with MWh, drafted this paper. All authors commented on the manuscript and approved the final draft prior to publication. All authors are guarantors for this work.

## Pre-publication history

The pre-publication history for this paper can be accessed here:


